# Polygenic Scores and Parental Predictors: An Adult Height Study Based on the United Kingdom Biobank and the Framingham Heart Study

**DOI:** 10.3389/fgene.2021.669441

**Published:** 2021-05-21

**Authors:** Chong You, Zhenwei Zhou, Jia Wen, Yun Li, Cheng Heng Pang, Haoyang Du, Ziwen Wang, Xiao-Hua Zhou, Daniel A. King, Ching-Ti Liu, Jie Huang

**Affiliations:** ^1^Department of Biostatistics, School of Public Health, Peking University, Beijing, China; ^2^Department of Biostatistics, Boston University, Boston, MA, United States; ^3^Department of Genetics, University of North Carolina at Chapel Hill, Chapel Hill, NC, United States; ^4^Department of Biostatistics, University of North Carolina at Chapel Hill, Chapel Hill, NC, United States; ^5^Faculty of Science and Engineering, University of Nottingham Ningbo, Ningbo, China; ^6^Department of Computer Science, School of Art and Science, Wake Forest University, Wake Forest, NC, United States; ^7^Department of Bioengineering, School of Engineering, Rice University, Houston, TX, United States; ^8^Department of Medicine, Stanford University, Palo Alto, CA, United States; ^9^Department of Global Health, School of Public Health, Peking University, Beijing, China; ^10^Institute for Global Health and Development, Peking University, Peking, China

**Keywords:** adult height, prediction, polygenic score, parental height, model selection

## Abstract

Human height is a polygenic trait, influenced by a large number of genomic loci. In the pre-genomic era, height prediction was based largely on parental height. More recent predictions of human height have made great strides by integrating genotypic data from large biobanks with improved statistical techniques. Nevertheless, recent studies have not leveraged parental height, an added feature that we hypothesized would offer complementary predictive value. In this study, we assessed the predictive power of polygenic risk scores (PRS) combined with the traditional parental height predictors. Our study analyzed genotypic data and parental height from 1,071 trios from the United Kingdom Biobank and 444 trios from the Framingham Heart Study. We explored a series of statistical models to fully evaluate the performance of several PRS constructed together with parental information and proposed a model we call PRS++ that includes gender, parental height, and PRSs of parents and proband. Our estimate of height with an *R*^2^ of ∼0.82 is, to our knowledge, the most accurate estimate yet achieved for predicting human adult height. Without parental information, the *R*^2^ from the best PRS-driven model is ∼0.73. In summary, using adult height prediction as an example, we demonstrated that traditional predictors still play important roles and merit integration into the current trends of intensive PRS approaches.

## Introduction

The prediction of human height has long been of great interest to the medical research community and as a model for complex trait prediction. During the 20th century, much of the adulthood height prediction has been based on parental information ref. (Wright and Cheetham, 1999), crudely estimated by doubling the height attained by age 2 or 18 months, for boys and girls, respectively. Over the past 20 years, with the emerging development and application of single nucleotide polymorphism (SNP) array technology, genetics has been widely explored to understand the underlying biology and to improve the prediction of adult height. First, in terms of variance explained and SNP heritability, three papers demonstrated increasing values: (Wright and Cheetham, 1999) in 2010, 45% of variance was explained by ∼300,000 common SNPs ref. (Yang et al., 2010) in 2015, 56% of variance was explained by ∼17 million imputed variants ref. (Yang et al., 2015); and (Yang et al., 2015) in 2017, 68.5% of SNP heritability based on the United Kingdom Biobank was estimated in ref. (Ge et al., 2017). Then, in terms of observed prediction accuracy, the squared correlation between phenotype and predictor (*R*^2^) ranges from 0.17 ref. (Wood et al., 2014) to 0.19 (0.442) ref. (Yengo et al., 2018) and 0.53 ref. (Lippert et al., 2017). A large R2 from the Lippert et al. study could be attributed to two factors: (1) inclusion of gender in the prediction model; (2) based on a cohort of participants of diverse ancestry.

A major limitation of the genomics studies above is the absence of parental height in the prediction model. In this study, we use trio data from two flagship population cohorts, the United Kingdom Biobank (UKB) Prospective cohort and the Framingham Heart Study (FHS), to evaluate the predictive power of conventional predictors (based on height of parents and gender of proband) and polygenic risk score (PRS). We aim to identify a more accurate method for the prediction of height.

## Materials and Methods

### Study Population

The United Kingdom Biobank^1^ is a large, prospective population-based cohort study that enrolled approximately 500,000 participants aged 40 to 69 between 2006 and 2010 ref. (Sudlow et al., 2015). The study has collected a multitude of phenotypic data from questionnaires, physical and biological measurements, and electronic health records as well as genome-wide genotype data. We used the KING software to identify parent–child pairs ref. (Manichaikul et al., 2010; Bycroft et al., 2018). We excluded pairs where the parent is less than 18 years older than the child. We then grouped the pairs into father–child pairs, mother–child pairs, and mother–father–child trios. Because the predictor PRS was based on samples of European ancestry, we only included trios where all members are self-reported white British. In total, we identified 1,017 trios of European ancestry.

The FHS cohort was used for external validation. The FHS cohort is a large longitudinal cohort consisting of parents and children initiated in 1948 to study chronic vascular disease, and subsequently other phenotypes, in the United States. It is a multigenerational community-based cohort that includes third-generation participants ref. (Tsao and Vasan, 2015). We randomly selected one offspring from each of the family available and identified 444 independent trios in total. Table 1 summarizes the characteristics of study participants.

**TABLE 1:** Characteristics of study participants.

Cohort	Subject	*N*	Age	Height	BMI
UKB	Proband-male	418	42.22 (1.8)	178.12 (6.56)	27.61 (4.3)
	Proband-female	599	42.48 (18.6)	165.01 (6.01)	25.72 (5.13)
	Father	1,017	66.8 (2.05)	174.07 (6.3)	28.14 (3.98)
	Mother	1,017	65.34 (2.26)	160.2 (5.82)	27.92 (4.9)
FHS	Proband-male	201	36.99 (8.63)	175.43 (6.03)	27.54 (5.01)
	Proband-female	243	37.45 (8.65)	162.14 (5.9)	25.94 (5.66)
	Father	444	38.33* (8.06)	173.27 (6.55)	24.07 (4.62)
	Mother	444	36.25* (7.67)	159.42 (5.84)	23.67 (4.18)

*The parents of FHS trios are in the second generation while the probands are in the third generation. The age is based on examination date. The examination date differs significantly between the two generations.

### Construction of Height PRS

We used summary statistics of two prominent height GWAS studies as allelic scoring references to construct the PRS. The first GWAS reported 697 SNPs in year 2014, denoted as GWAS-2014. The second GWAS is a meta-analysis between GWAS-2014 and UKB GWAS that reported 3,290 SNPs, and we denote this as GWAS-2018 ref. (Yengo et al., 2018). We denote PRS generated from these two GWAS as PRS.0 (697 SNPs) and PRS.1 (3290 SNPs), respectively. We then applied more liberal thresholds based on GWAS-2018 to include many more marginal significant SNPs that are expected to increase the prediction power of PRS ref. (Purcell et al., 2009; Inouye et al., 2018). We used a widely accepted P+T approach to pick independent SNPs whose *p* value is lower than 1e-02, 1e-03, 1e-04, 1e-05, 1e-06, 1e-07, and 1e-08, and we denoted these PRS as PRS.2, PRS.3, PRS.4, PRS.5, PRS.6, PRS.7, and PRS.8, accordingly. Given that GWAS-2018 includes some of the ∼3,000 samples used for this study, we ran another UKB-only height GWAS without these ∼3,000 samples and meta-analyzed it with the GIANT-2014 results, denoting this new meta-analysis as GWAS-2018b. We then generated a new set of PRS.2 to PRS.8 using the GWAS-2018b set as a reference, but we found that all seven PRS were extremely similar, with *r*^2^ > 0.99, suggesting essentially no complementarity. We kept the PRS derived from GWAS-2018, since it is publicly available. We first used PLINK software ref. (Purcell et al., 2007) to prune the full GWAS summary statistics to pick independent SNPs, based on a LD reference panel of the 1,000 genome project phase III. The pruning parameters were: 1 Mb sliding window and LD *r*^2^ of 0.1. We then used the PLINK “–score” function to calculate a PRS, based on the pruned list of SNPs. Both steps were run for each chromosome in parallel. Finally, we added up all the PRS for each chromosome into a single genome-wide PRS.

### Model Predictors

In this work, we included sex, age, the first 40 genomic principal components (PC1–PC40, see ref. (Bycroft et al., 2018) as an example of using first 40 PCs in phenotyping–genomic data analysis), 9 PRSs (PRS.0, PRS.1, and PRS.2-8), as well as parental height, parental age, the first 40 PCs of parents, and 9 parental PRSs as potential predictors. The genomic principal components were obtained based on the UKB genotyped variants using a set of 407,219 unrelated, high-quality samples and 147,604 high-quality markers ref. (Tsao and Vasan, 2015). In total, there were 153 potential predictors that may potentially affect the value of height. Identification of the most accurate and parsimonious prediction model is accomplished by the model selection approaches. In other words, we used a “hypothesis-free” approach and let the data determine which predictors should go or stay.

### Model Estimation

The model with the highest *R*^2^_cv_ averaged over 50 CV-sets in the UKB data was chosen as the best prediction model and was validated using FHS to show the generalizability of the model. See Appendix for more details on model selection and estimation, and the code is available on https://github.com/jielab/height.

## Results

Table 2 shows the number of pruned SNPs for PRS.2-8, and the Pearson correlation between height and each PRS. The highest correction is with PRS.2 (*r* 0.47).

**TABLE 2:** Characteristics of nine polygenic risk scores (PRS).

PRS	Selection criteria	Number of SNPs	Correlation with height (*r*)
PRS.0	*P* < 5E-08 (2014)	697	0.34
PRS.1	*P* < 5E-09 (2018)	3,290	0.38
PRS.2	*P* < 5E-02 (2018)	30,615	0.47
PRS.3	*P* < 5E-03 (2018)	18,432	0.45
PRS.4	*P* < 5E-04 (2018)	12,657	0.44
PRS.5	*P* < 5E-05 (2018)	9,410	0.43
PRS.6	*P* < 5E-06 (2018)	7,349	0.42
PRS.7	*P* < 5E-07 (2018)	5,920	0.41
PRS.8	*P* < 5E-08 (2018)	4,932	0.41

*All SNPs are independent, either based on the published paper or based on PLINK pruning methods used in this study. “(2014)” refers to ref. (Wood et al., 2014) and “(2018)” refers to ref. (Yengo et al., 2018).

The optimal model is as follows,

(1) $$\begin{array}{c} \mathrm{height}=\mathrm{sex}+\mathrm{PRS}.2+\mathrm{height}.m+\mathrm{PRS}.2.m+\mathrm{height}.f\\ +\mathrm{PRS}.2.f+\varepsilon \end{array}$$

we call a PRS++ model, where **height.m** and **height.f** denote the heights of mother and father, respectively, **PRS.2.m** and **PRS.2.f** denote the PRS of mother and father, respectively, and ε is a random error term in the model that captures measurement errors, such as variation of height across different time in the day. Of note, the average *R*^2^_cv_ of PRS++ over 50-fold cross-validation in UKB data was ∼0.82, which is higher than any other study that has been reported to our knowledge. To quantify the additional benefit of including parental information in the model, we removed the parental contribution from the model and observed a decrease in the average of *R*^2^_cv_ from ∼0.82 to ∼0.73, see more comparisons of different models in Table 3.

**TABLE 3:** The *R*^2^ of different models including PRS++.

Model	In-sample *R*^2^ in UKB	*R*^2^_cv_ over 50-fold in UKB	In-sample *R*^2^ in FHS	Out-of-sample *R*^2^ in FHS
Sex + PRS.2	0.7034	0.7135 (0.09)	0.7193	0.707
Sex + Parental height	0.7353	0.7517 (0.08)	0.7179	0.7093
Sex + PRS.2 + Parental height	0.7825	0.7890 (0.10)	0.7828	0.7678
Sex + PRS.2 + Parental height + Parental PSR.2s	0.8025	0.8150 (0.05)	0.8113	0.7866
Full Model	0.8317	0.7733 (0.07)	0.8242	–^∗^

*The full model out-of-sample R^2^ in FHS is not provided as some of the potential covariates in UKB are not available in FHS data.

**FIGURE 1:**
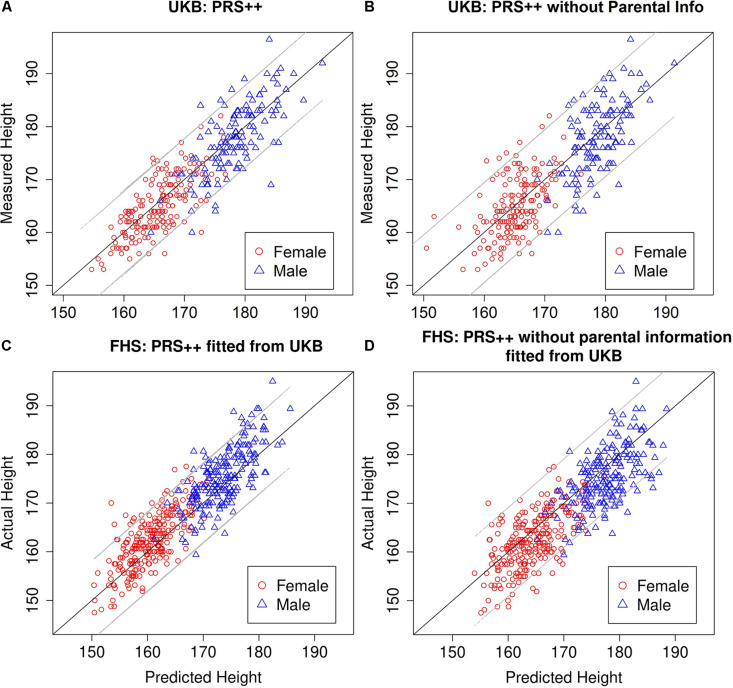
Measured vs. predicted heights of probands. Based on 300 randomly selected individuals from the United Kingdom Biobank trio data not included in predictor training in the PRS++ model **(A)**, the PRS++ model without parental information **(B)**, FHS using the PRS++ model fitted from United Kingdom Biobank data **(C)**, and FHS using the PRS++ model without parental information fitted from United Kingdom Biobank data **(D)**. The gray lines represent a 95% prediction interval.

The external validation set was built by testing the PRS++ model trained from the United Kingdom biobank data on the FHS data. The out-of-sample *R*^2^ based on the PRS++ model estimates from the United Kingdom biobank data was 0.7866, while the one without parental contribution was 0.7070. The in-sample *R*^2^ of PRS++ in FHS data was 0.8113. Such a difference in *R*^2^ was possibly due to the different population stratifications in two datasets as all participants are self-reported white British in our UKB data while FHS took place in the United States. Nonetheless, the inclusion of parental information increases the accuracy of the height prediction (see Figure 1 and Table 3).

## Discussion

Our study is the first to evaluate the predictive value of adult height, using all possible variables including parental height, the proband’s age and sex, genetic principal components, and millions of individual SNPs. By incorporating parental height, we were able to examine how “family history” incrementally improves height prediction beyond traditional factors such as age and sex and genetic factors such as PCs and even millions of significantly and marginally associated SNPs. We demonstrated the power of combining all these together to reach the most accurate prediction of adult height to our knowledge yet identified, with an *R*^2^ of 0.82. We note that the parental PRS.2s are included in the proposed PRS++ model. We speculate that the reason the parental PRSs are effective with the presence of parental height is that the parental height is in composite of known and unknown exposures, such as environmental effects and dining habit, rather than additive genetic effects.

There are a few limitations of this study. First, the sample size is small as the United Kingdom Biobank only contained approximately ∼1,000 informative trios. Second, the structure of the pedigree is simple. While we only examined height in trio structure, ideally, it might significantly increase model power if we could identify families with data from both parents and multiple offspring, especially those pedigrees where siblings have significantly different height. Third, given that height at childhood is prognostic for height at adulthood ref. (Wit and Oostdijk, 2011), the model accuracy may be improved by incorporating height at childhood, but we did not have access to those data. Other variables to consider for future study include status of certain diseases and lifestyle factors such as nutrition and exercise, or biomarker measurements such as growth hormone levels. Fourth, some studies adjust the phenotype for age and gender prior to the prediction analysis rather than including them as predictors. Their idea is that phenotype prediction is to see how well a phenotype can be predicted for people in the same age, gender and ancestral group. Therefore, the R2 reported in our study would not be directly comparable to such studies because our study did include age and gender as predictors.

In order to exclude disease as a confounder to our height estimates, such as chronic illness, or congenital disorders, we confirmed that none of the ∼3,000 samples included in our study have aneuploidy. Of note, the offspring included in the study cohort aged between 40 and 49, so that the growth would have been completed while the height loss due to aging would still be negligible.

In summary, our analyses demonstrated that parental height contributes to predicting adult height, even after accounting for a predictive value of >30,000 SNPs. Therefore, it is crucial for future statistical models to consider parental phenotypes that may potentially capture the missing heritability better, for example, Alzheimer’s disease, obesity, and cardiovascular disease.

## Data Availability Statement

The raw data is available on application to UK Biobank.

## Ethics Statement

UK Biobank had obtained ethics approval from the North West Multi-centre Research Ethics Committee which covers the UK (approval number: 11/NW/0382) and had obtained informed consent from all participants.

## Author Contributions

CY and JH designed, analyzed, and wrote this study. ZZ verified the model in the FHS data. DK improved the language and provided helpful suggestions. The rest authors provided helpful suggestions and proofread the text. All authors contributed to the article and approved the submitted version.

## Conflict of Interest

The authors declare that the research was conducted in the absence of any commercial or financial relationships that could be construed as a potential conflict of interest.

## Footnotes

1 http://www.ukbiobank.ac.uk
