# Supplementary figures and images for: Polygenic Scores and Parental Predictors: An Adult Height Study Based on the United Kingdom Biobank and the Framingham Heart Study
Source: Front Genet. 2021 May 21;12:669441. doi: 10.3389/fgene.2021.669441 (PMC8176283; doi:10.3389/fgene.2021.669441)

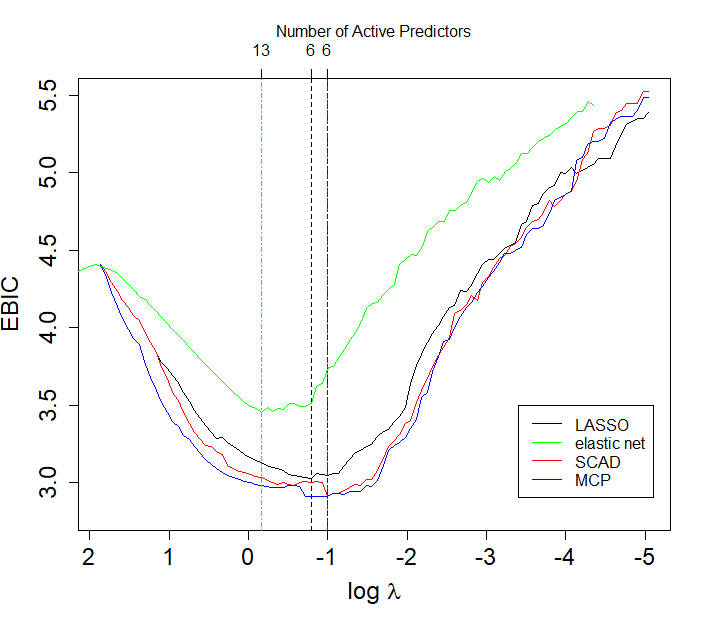

Supplement: Supplementary file 1 [file Image_1.PNG]

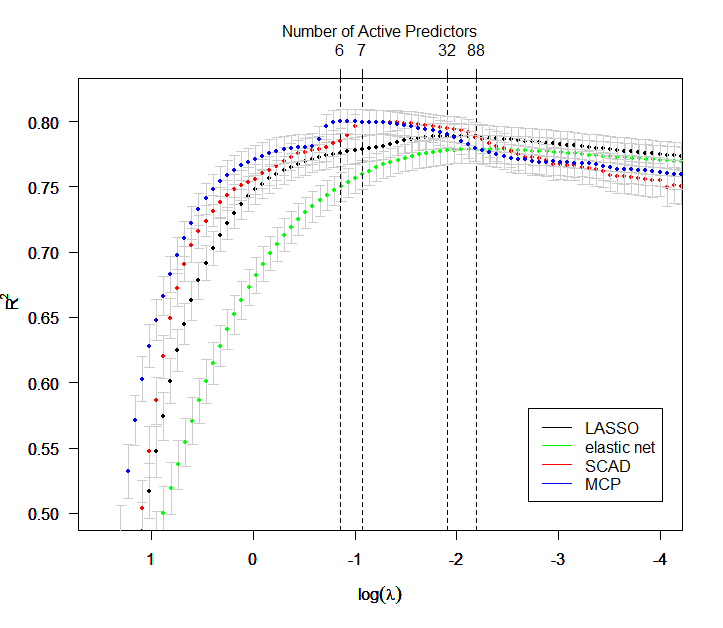

Supplement: Supplementary file 2 [file Image_2.PNG]

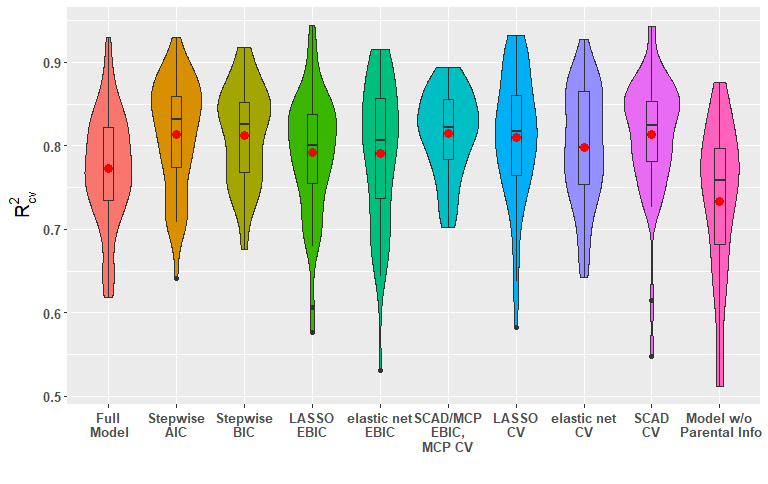

Supplement: Supplementary file 3 [file Image_3.PNG]
