# Supplementary material for: Polygenic Scores and Parental Predictors: An Adult Height Study Based on the United Kingdom Biobank and the Framingham Heart Study
Source: Front Genet. 2021 May 21;12:669441. doi: 10.3389/fgene.2021.669441 (PMC8176283; doi:10.3389/fgene.2021.669441)
Supplement: Supplementary file 6 [file Data_Sheet_1.docx]

**Supplementary Material**

**Model selection and estimation**

We used cross-validation (CV) to evaluate held-out predictions of candidate statistical models which were measured by the fraction of trait variance explained by the predictive model (R^2^_cv_), averaged over independent CV sets. We then tested the model using the Framingham dataset as external and completely independent dataset. Since the number of initial possible models is 2^153^ we limited the model selection space by best models from the following approaches: (1) step-wise AIC/BIC (ref. 1-2); (2) penalized regression methods implemented by the R package ncvreg, including LASSO, elastic net, SCAD and MCP, with the tuning parameter determined by the lowest extended BIC (EBIC) which has been shown to perform well in several contexts, and all other settings set at the default (ref. 3); and (3) the same penalized regression methods but using a tuning parameter with the highest average R^2^_cv_ over the 10-fold CV.

Penalty-based procedures impose direct constraints on regressors in the estimation procedure to encourage sparsity. Therefore, for penalty-based procedures, coefficient **estimation and** variable **selection** are performed **simultaneously**. However, it is known that the tuning parameter in penalized regression enforces shrinkage, and hence, inducing bias for the estimates of non-zero coefficients in the model. To reduce such bias, we re-fitted the selected models by penalty-based procedures using least square estimates. The selected models from the preliminary selection round were then evaluated by CV and the model with highest R^2^_cv_ averaged over 50 CV-sets was chosen as the best prediction model.

**Fig S1** displays the EBIC as a function of the tuning parameters $\lambda$ in LASSO, elastic net, SCAD and MCP regressions for height. The optimal tuning parameter is determined by the lowest EBIC. The axis on top of the plot denotes the number of active predictors in the model at that value of the tuning parameter $\lambda$ while the vertical line is placed at the value where the EBIC is minimized. The selected model by SCAD was identical to the one by MCP, which only included sex, PRS.2, parental heights and parental PRS.2, and was more parsimonious than that of LASSO and elastic net as expected. **Fig S2** shows the results from LASSO, elastic net, SCAD and MCP regressions with the tuning parameter determined by 10-fold cross validation. The y-axis in **Fig S2** represents the average R^2^_cv_ in the external validation dataset. The error bars reflect 1 SD of uncertainty estimated from ten replicates. The top axis denotes the number of active predictors in the model at that value of $\lambda$ while the vertical line is placed at the value where R^2^_cv_ is maximized. In general, the selected models based panelized regression with EBIC were more parsimonious than what were selected based panelized regression with CV. The stepwise BIC selected a very similar model to MCP regression using 10-fold CV while the stepwise AIC picked a least parsimonious model among the all. All the selected model contained sex, PRS.2 and parental heights as active predictors. See Table S1 for predictors included in optimal models based on different selection methods aforementioned. The two metrics for prediction accuracy in the table are R^2^ and (mean absolute difference) MAD.

The selected models were then compared using 50-fold CV (**Fig S3**). Model evaluation using 50-fold CV identified that the model selected by SCAD/MCP with EBIC had the highest average R^2^_cv_ of 0.81, and thus selected as the global optimal linear model:

**height ~ sex + PRS.2 + height.m + PRS.2.m + height.f + PRS.2. f (1)**,

which supports that the inclusion of parental information does improve the prediction accuracy at least in the linear model. Note that in terms of MAD, the above model is not the best model in Table S2, but the difference is small and the proposed model in (1) is more parsimonious.

Despite the main aim of the work is to assess if the inclusion of parental height yields a better prediction on height as compare to using linear model with GRS as predictors alone from many existing studies. It would be interesting to see the performances of non-linear models with and without parental information, for example, random forest. We have now conducted a random forest-based analyses, see Table S1. For a standard random forest approach, variable selection is not implemented (only ranks by how well they improve the purity of the node are given, but the one with low rank does not necessarily mean that its effect is not significant). Hence instead of giving the optimal model based on the random forest-based analysis as we did in the linear model-based analysis, we calculated R^2^ and MAD of the five candidate models by the default setting in *R* platform. The results in Table S2 again support that the model with parental information provides better accuracy. In addition, compare to the results from linear models in Table 3, PRS++ is better both in term of R^2^ and MAD.

**Reference**

1. Akaike H. Information theory and an extension of the maximum likelihood principle. In Proceedings of the Second International Symposium on Information Theory 1973; Akadémiai Kiadó, Budapest, 267-281.
2. Schwarz G. Estimating the dimension of a model. The Annals of Statistics 1978; 6(2): 461-464.
3. Wang H. Forward regression for ultra-high dimensional variable screening. Journal of the American Statistical Association 2009; 104 (488): 1512-1524.

**Figure S1.** The extended BIC as well as the number of active predictors are plotted as functions of the log of the tuning parameter $\lambda$ in the three penalized regressions. The vertical lines represent the best models chosen by lowest extended BIC in each of the penalized regression.

**Figure S2.** Prediction with the tuning parameter determined by 10-fold cross validation. The average R^2^ between actual and predicted over independent 10 folds as well as the number of active predictors are plotted as functions of the log of the tuning parameter in the three penalized regressions along with standard error bars. The vertical lines represent the best models chosen by 10-fold CV.

**Figure S3.** Prediction with the tuning parameter determined by 50-fold cross validation. The models are shown in the X-axis, which includes full model, optimal models in the preliminary selection step and the best model without parental information. The dots in the plot are the mean value of R^2^_cv_ for each model. The model selected by SCAD/MCP with EBIC and MCP by cross validation provides the highest average value of R^2^_cv_ in 50 independent folds.

**Table S1** Predictors included in optimal models selected by different model selection approached and the corresponding R^2^ and MAD in the UKB data.

**Table S2** Performance of various models based the random forest analysis.
